# Dye-Staining Angioscopy for Coronary Artery Disease

**DOI:** 10.1007/s12410-015-9327-z

**Published:** 2015-02-27

**Authors:** Yasumi Uchida, Yasuto Uchida

**Affiliations:** 1 Japanese Foundation for Cardiovascular Research, 2-30-17, Narashinodai, Funabashi, 274-0063 Japan; 2 Department of Cardiology, The Jikei University School of Medicine, Tokyo, Japan; 3 Department of Cardiology, Tsukuba Memorial Hospital, Tsukuba, Japan

**Keywords:** Angioscopy, Apolipoproteins, Biomarkers, Cardioscopy, Cholesterol, Lipoproteins, Myocardial tissue fluid flow, Subendocardial myocardial blood flow

## Abstract

Novel imaging techniques using biomarkers have clarified the mechanisms of hitherto unanswered or misunderstood phenomena of coronary artery disease and enabled evaluation of myocardial blood and tissue fluid flows in vivo. Dye-staining coronary angioscopy using Evans blue (EB) as the biomarker can visualize fibrin and damaged endothelial cells, revealing that the so-called platelet thrombus is frequently a fibrin-rich thrombus; occlusive transparent fibrin thrombus, but not platelet thrombus, is not infrequently a cause of acute coronary syndrome; “fluffy” coronary luminal surface is caused by fibrin threads arising from damaged endothelial cells and is a residue of an occlusive thrombus after autolysis in patients with acute coronary syndrome without angiographically demonstrable coronary stenosis; and web or membrane-like fibrin thrombus is a cause of stent edge restenosis. Fluorescent angioscopy using visual or near-infrared light wavelengths is now used clinically for molecular imaging of the substances such as lipoproteins and cholesterol that constitute coronary plaques. Dye-staining cardioscopy using EB or fluorescein enables direct and real-time visualization of subendocardial microcirculation.

## Introduction

Conventional coronary angioscopy (AS) is more useful for the evaluation of coronary plaque and thrombus than angiography, and cardioscopy (CS) is more useful for detecting intracardiac thrombi and myocardial diseases than ventriculography. However, both imaging modalities use visible light and both are limited to evaluating surface morphology and color of the target lesions, so it is beyond the scope of either to evaluate the composition of the target lesions. Coronary AS or CS using biocompatible markers is one choice for evaluation of the tissues, cells, or molecules that comprise the target lesions.

Evans blue (EB) is a blue dye used clinically for the measurement of cardiac output many years ago. Beneficial effects of this dye in preventing coronary restenosis have been reported [1, 2]. Histological examinations in animals and patients have revealed that EB stains fibrin and damaged vascular endothelial cells (ECs) [3].

In 1995, dye-staining AS was established and was used clinically to observe the peripheral arteries using EB as a biomarker for imaging fibrin and damaged ECs [4]. Further, this imaging technique was used to evaluate coronary, pulmonary, and aortic lesions in patients [5–7].

Furthermore, fluorescent coronary AS is now employed clinically for molecular imaging of the substances that constitute coronary plaques.

Dye-staining CS was established for evaluation of myocardial microcirculation [8–10].

Because these imaging techniques have been performed mainly in our laboratories, our imaging techniques and obtained findings are described here.

## Dye-Staining Coronary AS

### Coronary AS System and Its Manipulation

**Fig. 1:**
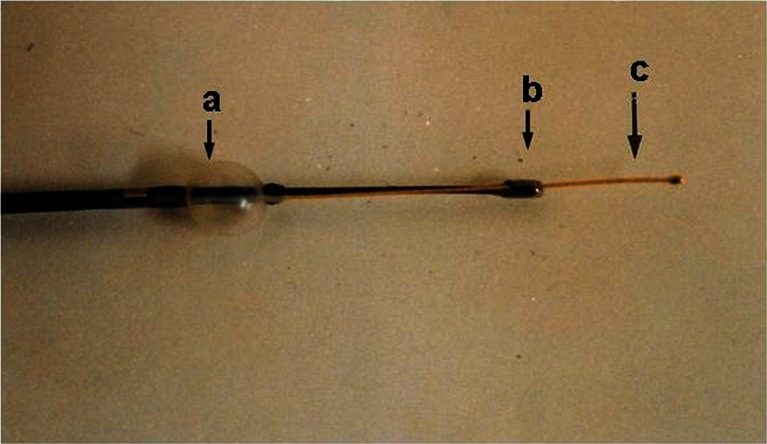
Monorail-type angioscope for coronary use. *a* Balloon. *b* Fiberscope. *c* Guidewire. Reproduced from ref. [3], with permission

The novel AS system comprises a light source (CTV-A, Olympus Corporation, Tokyo, Japan), an angioscope of the monorail type (VecMover, Clinical Supply Co., Gifu, Japan), and a color chilled charged device (CCD) camera (CSVEC-10, Clinical Supply) (Fig. 1) [11].

After coronary angiography, the angioscope is introduced into the targeted coronary artery. The balloon of the angioscope is inflated to stop the blood flow therein, and the fiberscope incorporated into the angioscope is slowly advanced up to 7 cm distally to facilitate successive observations of the artery while displacing the blood by infusion of heparinized saline solution (10 IU/mL) at a rate of 2 mL/s for 10–20 s through the flash channel of the angioscope. To accurately confirm the location of the angioscope tip (and accordingly the observed portion), the angioscopic and fluoroscopic images are displayed simultaneously on a monitor.

After the control observation, 1 mL of 2.5 % EB solution is injected during balloon inflation into the artery through the flush channel of the angioscope to stain damaged endothelial cells or fibrin, and then, the balloon is deflated to restore blood flow. At 1–2 min later, the balloon is re-inflated and the coronary luminal surface is observed by AS [12].

### Imaging of Coronary EC Damage Caused by Catheter Manipulation

Coronary ECs protect the vascular wall against spasm and thrombus formation through release of vasodilating and antithrombotic substances. When the ECs are damaged, thrombus immediately forms on that site [3]. However, it has been difficult to visualize damaged ECs in vivo.

**Fig. 2:**
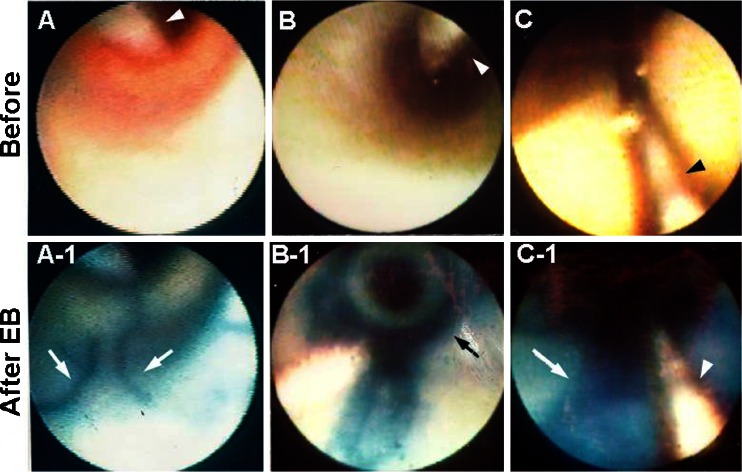
Visualization of coronary endothelial cell damage induced by catheter manipulation. *A* Conventional angioscopic image of a coronary segment after single introduction of a guidewire (*arrowhead*). *A-1* After injection of Evans blue (*EB*), linear endothelial cell damage caused by the guidewire (*arrows*) can be seen. *B* Conventional angioscopic image of a coronary segment after balloon inflation of an angioscope. *Arrowhead*: guidewire. *B-1* Dye-staining angioscopic image of the same portion. *Arrow*: circumscribed staining with EB, indicating balloon-induced endothelial cell damage. *C* Conventional angioscopic image of a coronary segment proximal to the target lesion treated by stent deployment. *Arrowhead*: guidewire. *C-1* After EB injection, the entire luminal surface stains blue, indicating extensive endothelial cell damage (*arrow*). *Arrowhead*: guidewire. Reproduced from ref. [3], with permission

Using dye-staining AS, damaged coronary ECs can be visualized, and it has become clear that cell damage is caused by insertion of a catheter for percutaneous intervention or balloon inflation and even by insertion of a guidewire (Fig. 2) [3, 13].

### Discrimination of Coronary Fibrin and Platelets in Patients with Acute Coronary Syndrome

**Fig. 3:**
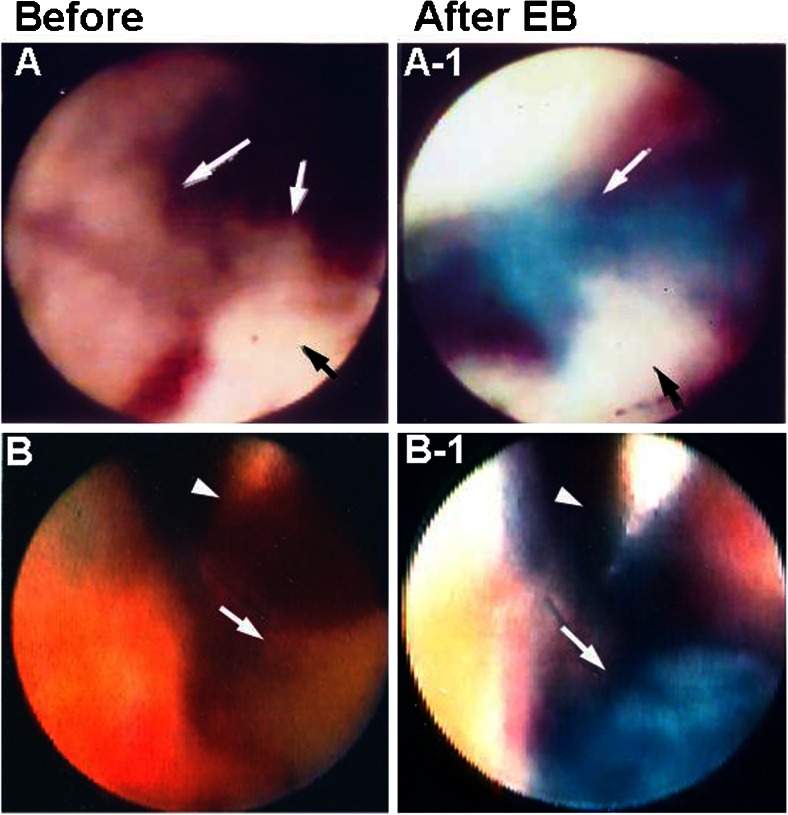
Discrimination of fibrin from platelets in coronary thrombi. *A* White coronary thrombi (*arrows*) in a patient with ST-elevation myocardial infarction. *A-1* After injection of Evans blue (*EB*), the area stained *blue* (*white arrow*) indicates the presence of fibrin, and the unstained area (*black arrow*) indicates platelet aggregates. *B* Brown mass (*arrow*) in the obstructed coronary segment in a patient with ST-elevation myocardial infarction. *Arrowhead*: guidewire. *B-1* The mass stained *blue* with EB, indicating a mixture of fibrin and plaque debris (*arrow*). *Arrowhead*: guidewire. Reproduced from ref. [12], with permission

Platelet thrombi play a key role in the genesis of acute coronary syndrome (ACS), and it has been generally considered that white thrombus is platelet thrombus [3]. Using dye-staining coronary AS and EB as a marker of fibrin to examine whether white coronary thrombus in patients with ACS is composed of platelets alone, it was revealed that the majority of white thrombi (so-called platelet thrombi) can be clearly discriminated into fibrin-rich and platelet-rich thrombi (Fig. 3) [12]. Therefore, the use of this imaging modality may contribute to selection of effective primary or adjunctive thrombolytic therapy.

### Identification of Platelet Thrombus

To date, there has not been an established imaging method to identify platelet thrombus in patients. My group found that fluorescein stains both fibrin and platelets (unpublished observation), so administering fluorescein after staining fibrin with EB may contribute to identification of platelet thrombus.

After confirming the existence of a thrombus in the culprit coronary segment in patients with ACS, EB is injected into the coronary artery. Next, 1 mL of 1 % solution of fluorescein is injected into the same artery, and the fluorescence is imaged with a fluorescent AS system using a band-pass filter of 470 nm and band-absorption filter of 515 nm. Details are described elsewhere [11].

**Fig. 4:**
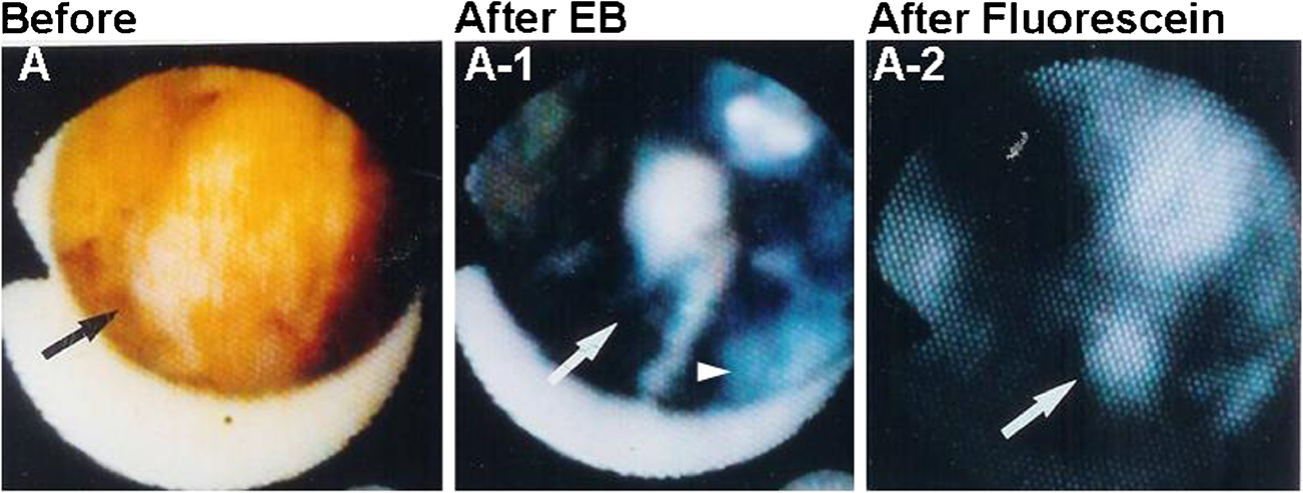
Identification of platelets by fluorescent angioscopy using fluorescein. *A* Brown coronary thrombus (*arrow*) observed by angioscopy before injection of Evans blue (*EB*) in a patient with ST-elevation myocardial infarction. *A-1* The thrombus partially stained *blue* with EB (*arrowhead*) and a *white area* remained (*arrow*). *A-2* After fluorescein injection, the white area fluoresced, indicating it comprised platelets (*arrow*)

Following fluorescein injection, the white portion of the thrombus, which does not stain with EB, exhibits fluorescence, indicating that it consists of platelets (Fig. 4).

### Angiographically Obstructed but Angioscopically Unobstructed Culprit Coronary Segment in Patients with ACS

**Fig. 5:**
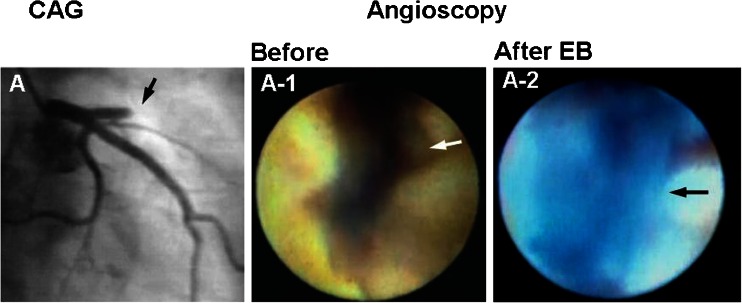
Angiographically obstructed but angioscopically unobstructed coronary segment in a patient with unstable angina. *A* Coronary angiography (*CAG*) showing proximal segment of the left anterior descending artery (*arrow*). *A-1* Angioscopic image of the angiographically obstructed segment (*arrow* in *A*). The segment was composed of disrupted plaque but a residual lumen existed (*arrow*). *A-2* After injection of Evans blue (*EB*), the residual lumen stained blue, indicating that it was obstructed with a transparent fibrin thrombus (*arrow*). Reproduced from ref. [14•], with permission

Figure 5 shows a patient with unstable angina (UA) in whom the left anterior descending artery was totally occluded according to angiography. However, a residual lumen was observed and nothing was seen in the lumen by conventional AS. Dye-staining AS exposed a blue structure occupying the residual lumen, indicating a fibrin thrombus causing total occlusion. Such transparent fibrin thrombi, namely a structure that is not visible with conventional AS and becomes visible with dye-staining AS, have been observed in patients with UA or non-ST elevation myocardial infarction (non-STEMI) but not in those with STEMI [14•].

### Detection of Damaged ECs on Coronary Stent Struts in the Chronic Phase

Coronary in-stent thrombosis is not infrequently observed by AS, even 6 months or more after stenting, despite the use of ticlopidine and aspirin. However, the mechanisms underlying late thrombosis are not well understood. ECs are highly antithrombotic, so it is possible that the mechanism is damage of the neo-ECs covering the stent struts.

**Fig. 6:**
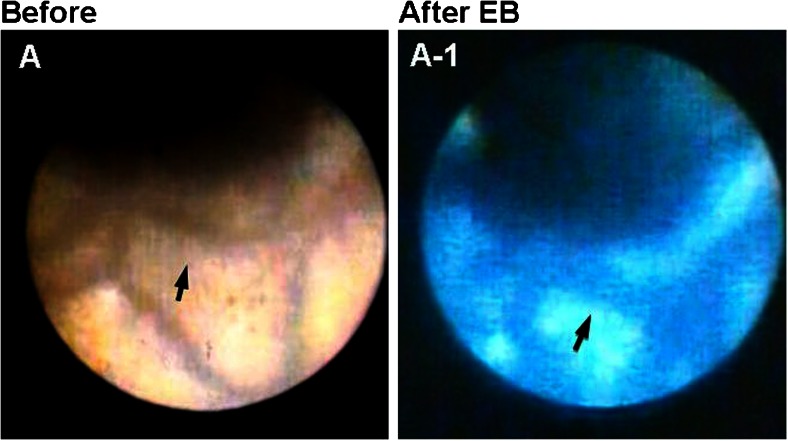
Staining of the endothelial cells covering stent struts in the chronic phase after stent deployment. *A* Stent struts can be seen through at 6 months after deployment of a Multilink stent (*arrow*). *A-1* After injection of Evans blue (*EB*), endothelial cells on the struts stained blue, indicating cell damage (*arrow*)

Angioscopic observation of the coronary segments 6 months after bare stent implantation was performed in 44 patients. Stent struts were classified by conventional AS into subgroups: not covered by neointima (naked group); visible through the neointima (see-through group); and not visible through the neointima (not-see-through group). EC damage visualized with EB was observed in 13.3 % of the not-see-through group and in 80 % of the see-through/naked group. The neo-ECs covering stent struts in the see-through/naked group stained blue with EB more frequently than in the not-see-through group, indicating neo-EC damage (Fig. 6), which was classified as localized or diffuse damage. Late stent thrombosis was observed in the latter type. In animals, late stent thrombosis occurs when the neointimal thickness is within 100 μm. Neo-ECs may be damaged by friction between them and the stent struts, because of a thin interposed neointima, which should act as a cushion, resulting in late stent thrombosis [15, 16]. These findings suggest the necessity of an appropriate thickness of neointima for prevention of late stent thrombosis. A similar mechanism may cause late stent thrombosis in patients in whom a drug-eluting stent is implanted.

### Fluffy Coronary Luminal Surface without Significant Stenosis or Obstructive Thrombus in Patients with ACS

**Fig. 7:**
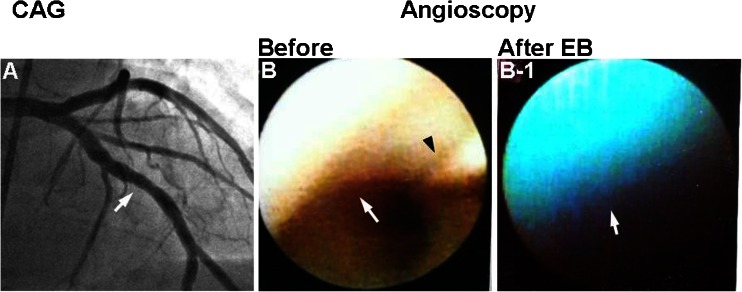
Fluffy luminal surface of culprit non-stenotic coronary segment in a patient with acute coronary syndrome. *A* Coronary angiogram (*CAG*) of left anterior descending artery. *Arrow*: Segment where fluffy luminal surface was observed by angioscopy. *A-1* Conventional angioscopy of the same segment, showing the “fluffy” luminal surface like seaweed (*arrow*). *Arrowhead*: guidewire. *A-2* After injection of Evans blue (*EB*), the fluffy luminal surface stained blue, suggesting the presence of fibrin threads (*arrow*). Reproduced from ref. [17], with permission

There are a considerable number of patients with ACS in whom no significant coronary obstruction is angiographically demonstrable. Hitherto, coronary spasm or accidental thrombosis has been considered as the underlying mechanism but without definite evidence. My group noticed a certain group of patients with ACS in whom significant stenosis is not demonstrable, and the suspect culprit coronary segment exhibits a “fluffy” (or frosted glass-like) surface. In a few of these patients, a thrombus distal to the fluffy segment is detected. The fluffy surface stains blue with EB, indicating the presence of fibrin and/or damaged ECs (Fig. 7).

Similar changes were reproduced by mechanical damage to the artery followed by blood perfusion to produce thrombus in dogs. Fluffy surface was exposed after removal of the globular thrombus. Histologically, the fluffy surface was composed of fibrin threads arising from the damaged ECs and adhered together by platelets. Therefore, it is proposed that a coronary segment with a fluffy or frosted glass-like surface is the site of surface disruption, and resultant thrombosis and the changes are related to residual fibrin and platelets after autolysis of the thrombus [17].

### Web and Membrane Formation on the Edges of Coronary Stents

Deployment of bare metal or drug-eluting stents into the coronary artery is widely performed to treat coronary artery disease (CAD). However, it has become evident that this effective therapeutic modality brings about the occurrence of several unwanted phenomena in patients, for instance in-stent restenosis and subacute or late stent thrombosis.

In the course of conducting routine evaluations of implanted stents using coronary AS, I and my colleagues frequently found band-like structures connecting two or more stent struts, namely a web and/or membrane-like structure connecting multiple stent struts like a curtain and obstructing the lumen. The incidence of this phenomenon was further investigated in the acute and chronic phases of the deployment of stents in patients suffering from ACS and from effort angina pectoris. Additional evaluation experiments were conducted in animals to obtain further clarification of the mechanisms entailed in this phenomenon.

**Fig. 8:**
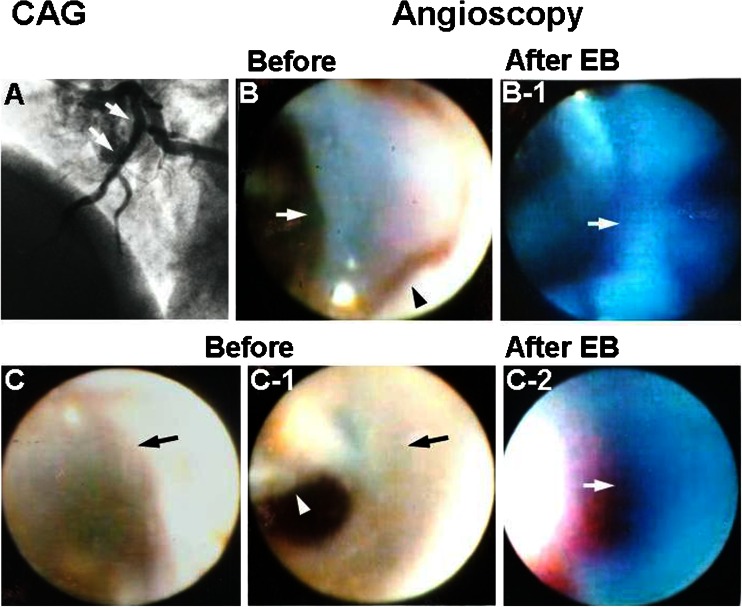
Web-like and membranous structures on the edges of stent. *A* Coronary angiography (*CAG*) immediately after bare metal stent deployment into the proximal segment of left anterior descending artery. *Arrows*: segment where angioscopy was performed. *B* Web-like structure on the proximal edge of the stent (*arrow*). *B-1* The web stained blue with Evans blue (*EB*), indicating it was composed of fibrin. *C* Membranous structure on the distal edge of the stent (*arrow*), easily displaced by a guidewire (*arrow*, *C-1*). *Arrowhead*: guidewire. *C-2* The membranous structure stained blue with EB, indicating it was composed of fibrin (*arrow*). Reproduced from ref. [17], with permission

Web and membrane were observed in both patients with ACS and those with effort angina (80.0 vs 18.7 %) immediately after stent deployment and also 6 months later (55.5 vs 28.5 %). The structures stained blue with EB immediately after stent deployment but not 6 months later. In beagles, the web and membrane were observed on stent edges in 75.0 % of cases at 5 h after stenting and in 66.6 % cases at 1 month later. Histological studies revealed that the web and membrane examined 5 h after stenting were composed of fibrin, whereas those examined 1 month later were composed of collagen fibers (Fig. 8) [18].

The results indicated that web and membrane frequently form on the edges of coronary stents and in the acute phase are composed of fibrin, which is replaced by collagen fibers in chronic phase. It is conceivable that these structures form because of blood flow turbulence and the affinity of stent struts for platelets/fibrin.

Those findings also indicated that pretreatment with heparin is not sufficient for prevention of web and membrane formation. Therefore, prophylactic fibrinolytic therapy just before stent deployment may be necessary to prevent this unwanted phenomenon.

Slow-flow phenomenon occurs in the stented coronary artery. Distal embolism and EC dysfunction have been proposed as the main causative mechanisms of this unwanted hemodynamic change [19]. Also, stent edge restenosis occurs in chronic phase [20]. There is a possibility that in addition to these mechanisms, the formation of web and membrane on the stent edges also contributes to the slow or no-flow phenomenon and stent edge restenosis.

## Fluorescent Angioscopy for Molecular Imaging of Coronary Plaques

Angioscopy which enables observation of the structural changes of the plaques and the substances which cause the plaque vulnerable was awaited for characterization of the vulnerable plaques.

Recently, fluorescent angioscopy was established, and they are now clinically used for imaging of coronary plaques.

By color fluorescent angioscopy using a band-pass filter of 345 nm and band-absorption filter of 420 nm, the yellow plaques observed by conventional angioscopy were further classified into three categories, namely green, white to light blue, and yellow to orange ones, and histological examinations revealed that the yellow to orange plaques lack collagen fibers, which protect the them against disruption, indicating that they are most susceptible to mechanical stress, namely vulnerable [21, 22].

**Fig. 9:**
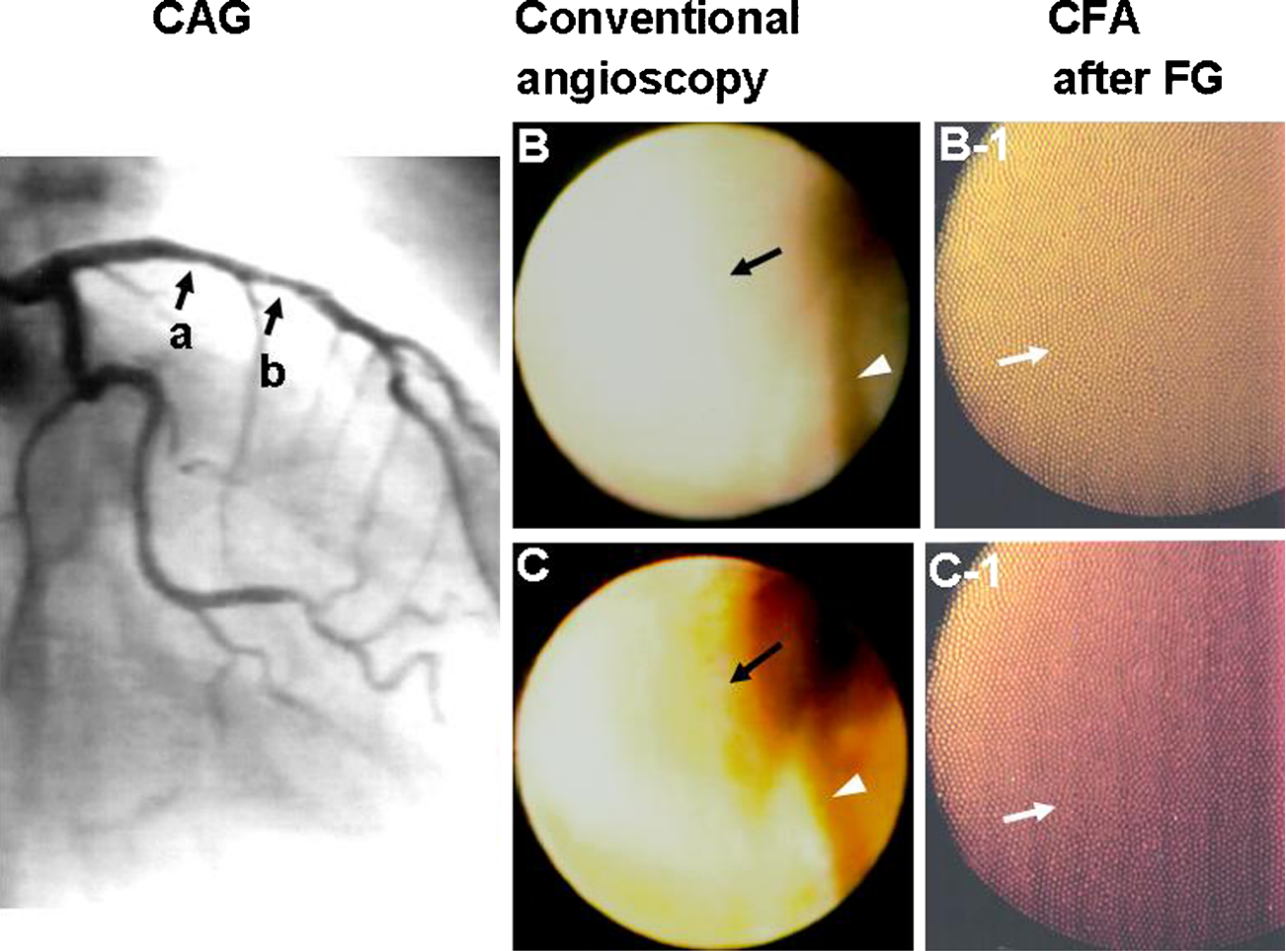
Imaging of HDL in human coronary plaques. Sixty-two-year-old male. *CAG* coronary angiography. *A* Angiogram of left coronary artery shows a slight stenosis in the proximal (*arrow a*) to middle segment (*arrow b*) of anterior descending artery. *B* Conventional angioscopic image of the portion indicated by *arrow a* in *A* shows *light yellow* plaque. *Arrowhead*: guidewire. *B-1* Color fluorescent angioscopic (*CFA*) image of the same portion after administration of Fast green dye (*FG*) shows diffuse brown fluorescence, indicating diffuse deposition of HDL (*arrow*). *B* Conventional angioscopic image of the portion indicated by *arrow b* in *A* shows a *yellow* plaque (*arrow*). *Arrowhead*: guidewire. *C-1* CFA image of the same portion after administration of FG shows *red* (*arrow*) fluorescence, indicating deposition of LDL and/or lysophosphatidylcholine. Reproduced from ref [26••], with permission

Oxidized low-density lipoprotein (oxLDL) and low-density lipoprotein (LDL), which play important roles in initiation, progression, and destabilization of coronary plaques, became visible in human coronary plaques by color fluorescent angioscopy using dyes as biomarkers [21, 23, 24]. Also, triglyceride and apolipoprotein B-100, which comprise LDL, also became visible [25]. Further, high-density lipoprotein (HDL) which counteracts with oxLDL became visible (Fig. 9) [26••, 27].

Imaging of cholesterol and cholesteryl esters, calcium, and LDL within 700 μm in depth from the plaque surface became visible by near-infrared fluorescent angioscopy using a band-pass filter of 685 nm and band-absorption filter of 780 nm [28, 29].

Among these, oxLDL, HDL, cholesterol, cholesteryl esters, and calcium were successfully imaged in patients with coronary artery disease in vivo.

Thus, this new imaging technologies may contribute clinically to clarify molecular mechanisms of coronary plaque growth and destabilization and to evaluate the effects on them of interventional and medical therapies.

## Dye-Staining CS

Structurally, the left ventricular wall of the heart comprises three myocardial layers, namely the inner oblique, middle circular, and outer oblique myocardial layers [30, 31]. It is the inner oblique layer that is most susceptible to ischemia. Until recently, myocardial blood flow has been evaluated by contrast echocardiography, radionuclide imaging, magnetic resonance imaging, computed tomography, or electron beam computed tomography. However, selective evaluation of the blood flow in the individual myocardial layers, especially in the inner oblique layer, namely the subendocardial myocardium, is often difficult with these imaging modalities.

A CS system using white light as the light source and obtaining color images of the heart from the inside, namely conventional CS, was devised [32–34] and used for differential diagnosis of myocardial and valvular diseases. This imaging modality also enabled observation of subendocardial myocardial blood flow (SMBF). However, because endocardial color was used as the indicator, assessment of SMBF was greatly influenced by the intensity of the light source. Therefore, a more reliable method for direct imaging of SMBF was required.

As the safety of intravascular administration of EB became evident from dye-staining AS studies, intracoronary administration was performed in patients with CAD to observe SMBF disturbance by CS using EB as an indicator, namely “dye-staining CS.”

### CS System and Its Manipulation

**Fig. 10:**
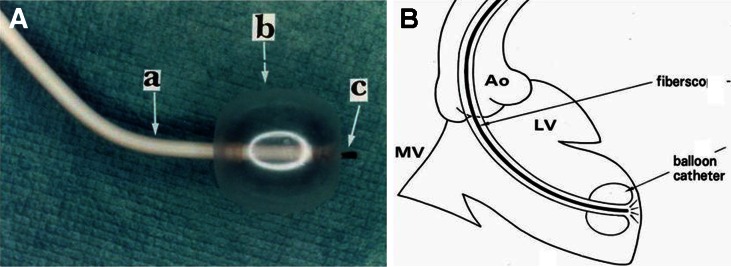
Cardioscopy system. *A* Cardioscope. *a* Guiding balloon catheter shaft. *b* Balloon. *c* Fiberscope. *B* Schematic of the method of observing the left ventricular endocardial surface with the cardioscope. *Ao* aorta. *MV* mitral valve. *LV* left ventricle. Reproduced from ref. [3], with permission

The CS system comprises a light source (CLV-A, Olympus), 9-F guiding balloon catheter (Clinical Supply), 4.2-F fiberscope (AF 14, Olympus), and a color CCD camera (OTV-A, Olympus) (Fig. 10).

Patients are pretreated with oral diazepam (10 mg) before being transferred to the catheterization laboratory. Left ventriculography is performed after administering 50 mg of intravenous lidocaine and 5,000 IU heparin. A 9-F guiding balloon is then introduced into the left ventricle and the balloon inflated with CO_2_. Next, a 5-F fiberscope is advanced through the catheter to position the fiberscope tip at the tip of the catheter. The balloon is gently pushed against the targeted wall segment of the left ventricle, and 50–100 mL of saline solution (heparin 10 IU/mL, 37 °C) is injected through the catheter at 10 mL/s to displace the blood between the balloon and the ventricular endocardial surface being observed. The anterior, apical, inferior, and lateral walls of the left ventricle are observed. The changes in the endocardial surface are recorded on a DVD recorder using the color CCD camera.

After the control observation, the balloon catheter is replaced by a Judkins catheter, and 1 mL of 2.5 % EB is injected into the coronary artery that irrigates the left ventricular wall segment under study. The balloon catheter and fiberscope are reintroduced into the left ventricle, placing the balloon catheter tip on the same wall segment that was previously observed, and the luminal surface changes are observed again [9].

### Evaluation of SMBF by Dye-Staining CS Using EB as a Biomarker

The observed portion is the anteroapical segment in chest pain syndrome; the wall segment that is irrigated by the artery in which spasm is evoked by intracoronary administration of acetylcholine in vasospastic angina (VSA); the wall segment irrigated by the stenotic artery in angina; and the dys- or akinetic wall segment in old myocardial infarction.

**Fig. 11:**
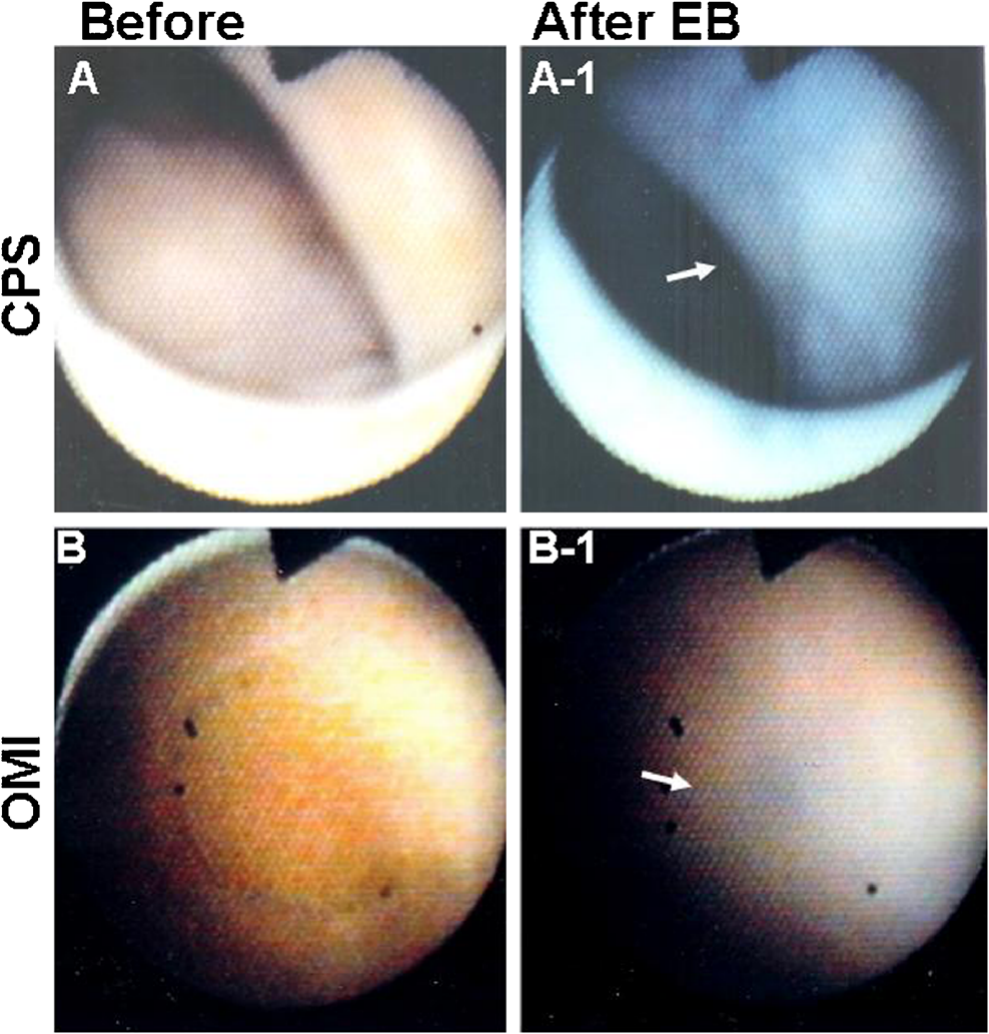
Dye-staining cardioscopy. *A*, *A-1* Patient with chest pain syndrome without significant coronary stenosis. *A* Before intracoronary injection of Evans blue (*EB*), the endocardial color was brown, indicating normal myocardial blood flow. *A-1* After intracoronary EB injection, the endocardial surface diffusely stained blue, indicating normal myocardial blood flow (*arrow*). *B*, *B-1* Patient with old myocardial infarction. *B* Endocardial surface is *white* to *yellowish brown. B-1* After intracoronary EB injection, the endocardial surface partially stained blue, indicating that myocardial blood flow remained in a small area (*arrow*). Reproduced from ref. [9], with permission

In patients without CAD, the endocardial surface diffusely stains blue immediately after intracoronary injection of EB, indicating normal SMBF in all patients with chest pain syndrome. In contrast, in patients with organic CAD, patchy staining indicating patchy preservation of SMBF and no staining indicating absent SMBF are frequently observed. In addition, patchy staining is also observed in patients with VSA, a functional disease (Fig. 11).

The effects of coronary stenting on SMBF have also been investigated, confirming restoration of SMBF by dye-staining CS in selected patients [9].

### Dye-Staining CS Using Fluorescein as a Biomarker for Evaluation of Myocardial Tissue Fluid Flow (MTFF)

MTFF plays a critical role in maintaining the function and survival of cardiomyocytes. The myocardial tissue fluid receives oxygen and nourishments from the coronary arterial capillaries and conveys them to the cardiomyocytes, receiving carbon dioxide and waste matter from the cardiomyocytes and conveying them to the venous capillaries. Therefore, it is important to measure the MTFF, but there are no clinically available tools to measure it in vivo.

Fluorescein is used clinically to evaluate retinal vessels [34]. Within the blood vessel, fluorescence of this dye is masked by the corpuscles, but when separated from these, the dye exhibits strong fluorescence [3]. When injected into the coronary vessels, it diffuses through the microvascular wall, separate from the blood corpuscles, into the interstitial spaces and exhibits strong fluorescence, representing tissue fluid flow. Therefore, if the fluorescence of this dye in the myocardium can be visualized in vivo, a real-time evaluation of MTFF can be attained.

My group developed a fluorescent CS system, and using fluorescein as an indicator, the left ventricular subendocardial MTFF was evaluated in patients with CAD. Furthermore, the effects on MTFF of percutaneous coronary intervention or vasodilating agents were evaluated.

#### Fluorescent CS System and Its Manipulation

The fluorescent cardioscopy system comprises a fluorescent excitation unit, angioscope, guiding balloon catheter, fluorescent emission unit, intensified CCD (ICCD) camera, camera controller, DVD recorder, and monitor.

The fluorescent excitation unit (CLV-A, Olympus) comprises a xenon lamp and a filter disc with a band-pass filter of 470 nm for fluorescence excitation. The fluorescent emission unit (DD-2, Olympus) comprises a dichroic membrane, which cuts the wavelength of light below 515 nm, a band-absorption filter of 515 nm, which allows a wave length of light more than 515 nm, an ICCD camera (C3505, Hamamatsu Photonics Co., Hamamatsu, Japan), and a camera controller (C3510, Hamamatsu Photonics).

The cardioscope and guiding balloon catheter are the same as those used for CS using EB. After conventional CS, the light and image guides are connected to the excitation and emission units, respectively. After setting the band-pass and band-absorption filters, the light is irradiated onto the target through the band-pass filter and light guide. The consequently evoked autofluorescence of the target is received by the ICCD camera through the dichroic membrane and band-absorption filters.

After a control observation, 3 mL of 10 % fluorescein (Fluoreside^R^, Japan Alcon Co., Tokyo), which is clinically used for imaging of blood flow [35], is injected into the right femoral vein. Fluorescent images are obtained at 0.5, 1, 3, and 6 min after finishing fluorescein injection [10].

#### MTFF in Patients with CAD

**Fig. 12:**
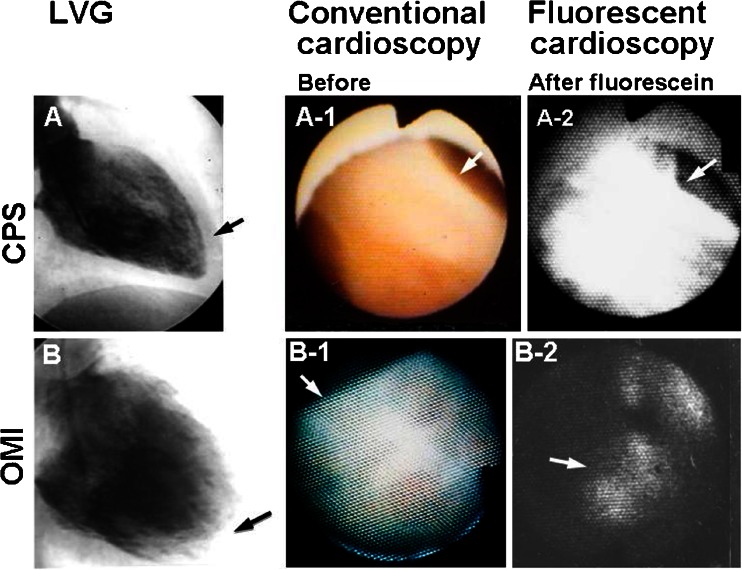
Cardioscopy using fluorescein as a biomarker (fluorescent cardioscopy). *A–A-2* Patient with chest pain syndrome without demonstrable coronary stenosis. *A* Left venticulogram shows normal contraction. *Arrow*: the portion observed by cardioscopy. *A-1* Conventional cardioscopy revealed normal brown color of the endocardial surface, indicating normal myocardial blood flow. *A-2* After intravenous fluorescein injection, the endocardial surface exhibited strong and diffuse fluorescence, indicating normal myocardial tissue fluid flow. *B* Left ventriculogram of a patient with old myocardial infarction (three-vessel disease). Left ventricular contraction was severely reduced. *Arrow*: the portion observed by cardioscopy. *B-1* The white endocardial surface indicates severe ischemia. *B-2* After fluorescein injection, the endocardial surface partially stained with fluorescein, indicating residual myocardial tissue fluid flow (*arrow*). Reproduced from ref. [10], with permission

The fluorescent images of MTFF are classified as follows: diffuse with high intensity indicating normal MTFF; diffuse but with low intensity indicating decreased MTFF; no fluorescence indicating absent MTFF; and patchy fluorescence indicating patchy preservation of MTFF. In a previous study, MTFF was normal in all 18 patients with chest pain syndrome, patchy, decreased, or absent MTFF in 16 of 20 patients with angina pectoris and/or old myocardial infarction from organic CAD, and patchy in 21 of 28 patents with VSA. In 10 of 20 patients who underwent coronary stenting, the angiographic result was successful, but MTFF disturbance frequently remained (Fig. 12) [10].

## Conclusions

Dye-staining AS using EB as a biomarker has clarified the mechanisms of hitherto unanswered or misunderstood phenomena of CAD by visualizing fibrin and damaged ECs; so-called platelet thrombus is frequently a fibrin-rich thrombus; occlusive transparent fibrin thrombus but not platelet thrombus is not infrequently a cause of ACS; fluffy coronary luminal surface is caused by fibrin threads attached to spontaneously damaged ECs and is a residue of occlusive thrombus after autolysis in patients with ACS and without angiographically demonstrable coronary stenosis; and web or membrane-like fibrin thrombus is a cause of stent edge restenosis, suggesting the necessity for fibrinolytic therapy for restenosis prevention.

Using new biomarkers, this imaging technique is now used clinically for molecular imaging of the substances that constitute atherosclerotic lesions. Dye-staining CS using EB enables direct and real-time visualization of SMBF and therefore enables determination of which coronary branch supplies collateral blood flow to the observed wall segment. Fluorescent CS using fluorescein as a biomarker enables visualization of real-time changes in MTFF which plays a critical role in maintaining the function and survival of cardiomyocytes.

## Abbreviations

AS: Angioscopy
ACS: Acute coronary syndrome
CCD: Chilled charged device
CS: Cardioscopy
EB: Evans blue
MTFF: Myocardial tissue fluid flow
SMBF: Subendocardial myocardial blood flow

## References

[1] Uchida Y. Therapeutic tool for vascular disease. US Patent No. 7,025,981 B2. Washington, DC: US patent and Trademark Office. Issued to Yasumi Uchida, 2006.

[2] Uchida Y. Medicines for treatment of atherosclerosis. Japanese Patent No. P. 153737A issued to Yasumi Uchida, 2007.

[3] Uchida Y, Uchida Y (2001). Physiological and histological basis for understanding blood stream, vascular endothelial damage, thrombosis, and thrombolysis. Coronary angioscopy.

[4] Uchida Y, Nakamura F, Tomaru T (1995). Observation of atherosclerotic lesions by an intravascular microscope in patients with arteriosclerosis obliterans. Am Heart J.

[5] Uchida Y (2011). Recent advances in coronary angioscopy. J Cardiol.

[6] Uchida Y, Uchida Y, Shirai S (2010). Angioscopic detection of pulmonary thromboemboli: with special reference to comparison with angiography, intravascular ultrasonography and computed tomography angiography. J Interv Cardiol.

[7] Uchida Y, Uchida Y (2012). Advances in angioscopic imaging of vascular disease. World J Cardiovasc Surg.

[8] Uchida Y (2011). Recent advances in percutaneous cardioscopy. Curr Cardiovasc Imaging Rep.

[9] Uchida Y, Uchida Y, Sakurai T (2010). Imaging of subendocardial blood flow by dye-staining cardioscopy in patients with coronary artery disease. Int Heart J.

[10] Uchida Y, Uchida Y, Kanai M (2010). Evaluation of myocardial tissue fluid flow by fluorescence cardioscopy in patients with coronary artery disease. Int Heart J.

[11] Uchida Y, Uchida Y (2001). Coronary angioscopy systems and their manipulation. Coronary angioscopy.

[12] Uchida Y, Uchida Y, Sakurai T (2011). Characterization of coronary fibrin thrombus in patients with acute coronary syndrome using dye-staining angioscopy. Arterioscler Thromb Vasc Biol.

[13] Terasawa K, Fujimori Y, Morio H (2000). Evaluation of coronary endothelial damages caused by PTCA guidewire: in vivo dye staining angioscopy. J Jpn Coll Angiol.

[14.•] Uchida Y, Uchida Y, Sakurai T (2011). Fibrin thrombus in unstable angina and NSTEMI. JACC Cardiovasc Imaging.

[15] Uchida Y, Uchida Y (2010). Angioscopic evaluation of neointimal coverage of coronary stents. Curr Cardiovasc Imaging Rep.

[16] Uchida Y, Uchida Y, Sakurai T (2011). Possible role of damaged neoendothelial cells in the genesis of coronary stent thrombus in chronic phase. Int Heart J.

[17] Uchida Y, Uchida Y, Sakurai T (2010). Fluffy luminal surface of the non-stenotic culprit coronary artery in patients with acute coronary syndrome. An angioscopic study. Circ J.

[18] Uchida Y, Uchida Y, Matsuyama A (2010). Formation of web- and membrane-like structures on the edges of bare-metal coronary stents. Circ J.

[19] Airoldi F, Buriguori C, Cianflone D (2007). Frequency of slow flow following stent implantation and effect of nitroprusside. Am J Cardiol.

[20] Jensen LO, Maeng M, Mintz GS (2009). Serial intravascular ultrasound analysis of peri-stent remodeling and proximal and distal edge effects after sirolimus-eluting or paclitaxel-eluting stent implantation in patients with diabetes mellitus. Am J Cardiol.

[21] Uchida Y, Uchida Y, Kawai S (2010). Detection of vulnerable coronary plaques by color fluorescent angioscopy. JACC Cardiovasc Imaging.

[22] Uchida Y, Maezawa Y, Schaller B (2012). Molecular imaging of atherosclerotic coronary plaques by fluorescent angiosocopy. Molecular imaging.

[23] Uchida Y, Maezawa Y, Uchida Y (2013). Localization of oxidized low-density lipoprotein and its relation to plaque morphology in human coronary artery. PLoS One.

[24] Uchida Y, Maezawa Y, Uchida Y (2012). Molecular imaging of low-density lipoprotein in human coronary plaques by color fluorescent angioscopy and microscopy. PLoS One.

[25] Hiruta N, Uchida Y, Maezawa Y (2013). Molecular imaging of apolipoprotein B-100 in human coronary plaques by color fluorescent angioscopy and microscopy. Int Heart J.

[26.••] Uchida Y, Uchida Y, Hiruta N (2013). Molecular imaging of native high-density lipoprotein in human coronary plaques by color fluorescent angioscopy. JACC Cardiovasc Imaging.

[27] Uchida Y, Hirta N, Uchida Y (2013). Localization of native high-density lipoprotein and its relation to plaque morphology in human coronary artery. Int Heart J.

[28] Uchida Y, Uchida Y, Sugiyama Y (2010). Two-dimensional visualization of cholesterol and cholesteryl esters within human coronary plaques by near-infrared fluorescence angioscopy. Clin Cardiol.

[29] Uchida Y, Uchida Y. Imaging of native low-density lipoprotein in human coronary plaques by near-infrared angioscopy. Proceedings of 28th Annual Meeting of Japanese Society for Cardioangioscopy, Oct 5, 2014; Nagoya, Japan, p. 12.

[30] Bhatis S, Levi M (1996). The pathology of congenital heart disease.

[31] Fujita T, Fujita T (1993). Myocardial anatomy. Human anatomy.

[32] Uchida Y, Nakamura F, Oshima T (1990). Percutaneous fiberoptic angioscopy of the left ventricle in patients with idiopathic dilated cardiomyopathy and acute myocarditis. Am Heart J.

[33] Uchida Y (1995). Atlas of cardioangioscopy.

[34] Uchida Y, Zipes DP, Rowlands BJ (1991). Percutaneous fiberoptic angioscopy of cardiac chambers and valves. Progress in cardiology.

[35] Albert DM, Jakobiec FA (1994). Fluorescein angiography. Principles and practice of ophthalmology.

